# When hemolysis masks polycythemia vera

**DOI:** 10.1002/ccr3.1776

**Published:** 2019-01-28

**Authors:** Coralie Derrieux, Roland Jeandel, Antoine Martin, Christine Dosquet, Bruno Cassinat, Loïc Fouillard

**Affiliations:** ^1^ Laboratoire d'hématologie Grand Hôpital de l'Est Francilien Meaux France; ^2^ Service d'anatomie et Cytologie Pathologiques Grand Hôpital de l'Est Francilien Jossigny France; ^3^ Département de Pathologie Hôpital Avicenne Bobigny France; ^4^ Service de Biologie Cellulaire Hôpital Saint‐Louis Paris France; ^5^ Service d'hématologie Clinique Grand Hôpital de l'Est Francilien Meaux France

**Keywords:** hemolysis, masked polycythemia vera, panmyelosis

## Abstract

Although uncommon, clinicians should be aware that polycythemia vera may be masked due to hemolysis. The report of such associations could help them in clinical practice to establish an early and accurate diagnosis that may be challenging in atypical presentations of myeloproliferative neoplasms.

## 1 INTRODUCTION

Polycythemia vera is a chronic myeloproliferative neoplasm (MPN) characterized by a constitutive activation of the JAK‐STAT pathway that lead to an erythropoietin‐independent erythroid growth, a panmyelosis and an increased red blood count.^1^ In approximately 97% of cases, a *JAK2* V617F mutation is detected whereas about 3% of patients present a *JAK2* exon 12 mutation.^2^ In 2016, the WHO criteria of PV have been revised to improve the sensitivity of the diagnosis. The thresholds for hemoglobin (Hb) levels have been decreased from 18.5 to 16.5 g/dL for male and from 18.0 to 16.0 g/dL for female and hematocrit (Ht) cutoffs have been introduced (49% for male and 48% for female), allowing the diagnosis of cases with mild increases in Hb and/or Ht levels.^3^ These cases likely correspond to early phases of the disease or to masked PV (mPV), as defined in several studies in the view of the 2008 WHO classification. Several definitions were used for mPV, and controversial results were observed between studies in terms of outcomes.^4^, ^5^, ^6^ We report here the case of a patient with a PV masked due to hemolysis, highlighting that some patients could still be undiagnosed despite the recent revision of the WHO criteria.^3^

## 2 CASE PRESENTATION

**Figure 1:**
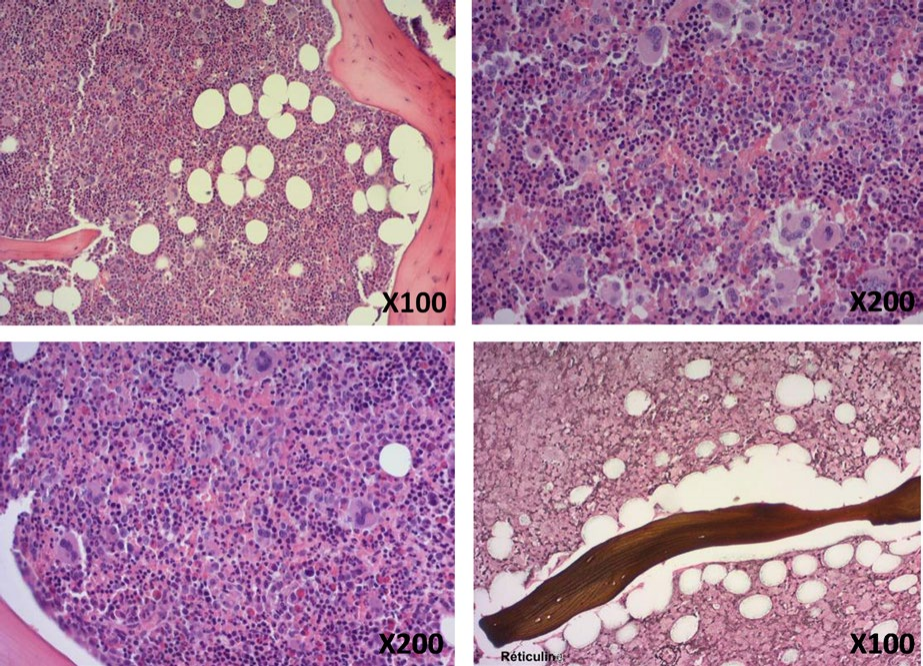
Histological features in the bone marrow biopsy. HES staining demonstrated a hypercellular bone marrow with a panmyelosis feature. Megakaryocytes were increased in number, pleiomorphic in size, without significant morphologic abnormalities, and sometimes regrouped in loose clusters (panels left upper and lower and right upper). No fibrosis was observed at reticulin staining (panel right lower)

A 59‐year‐old male presented a 3‐month history of white blood count (WBC) increase. Past medical history was not significant. Physical examination was unremarkable with a conserved general status and the absence of lymph node enlargement, splenomegaly, or hepatomegaly. An abdominal ultrasound revealed the presence of a mild splenomegaly. The full blood count demonstrated a WBC increase at 23.9 × 10^9^/L, including a neutrophilia at 20.8 × 10^9^/L, a mild basophilia at 0.5 × 10^9^/L and a slight eosinophilia at 0.7 × 10^9^/L. Normal Hb level (14.5 g/dL), Ht (44%), mean corpuscular volume (93 fL), and mean corpuscular hemoglobin concentration (33%) were observed. Platelet, lymphocyte, and monocyte count were in normal range (298 × 10^9^/L, 1.5 × 10^9^/L, and 0.5 × 10^9^/L, respectively). Interestingly, a high reticulocyte count at 227 × 10^9^/L was observed. The hypothesis of a MPN was established, and a peripheral blood (PB) molecular screening was performed. A *JAK2* V617F mutation was detected with a high mutated allele burden at 54%. An additional *EZH2* exon 8 mutation was detected by next‐generation sequencing (NGS). All other mutations screened, including *CSF3R* exon 14‐17 mutations, and BCR‐ABL1 transcripts were negative, ruling out the diagnosis of chronic neutrophilic leukemia and chronic myeloid leukemia, respectively. The complete list of the mutations screened by NGS is reported in Table 1. The presence of splenomegaly, leucocytosis with neutrophilia, and basophilia and a high *V617F JAK2* allele burden conduce to hypothesize a Primitive MyeloFibrosis (PMF) and a bone marrow (BM) biopsy was performed. Unexpectedly, the BM sections demonstrated hypercellulary with a panmyelosis feature, similar to that observed in PV. Megakaryocytes were increased in number, pleiomorphic in size, without significant morphologic abnormalities, and sometimes regrouped in loose clusters. No fibrosis was observed at reticulin staining (Figure 1). The BM smear was hypercellular with numerous megakaryocytes, an equilibrated granulocytic to erythroblastic ratio and a slight increase in basophilic cells, estimated at 2% (not shown). A normal 46,XY[25] karyotype was observed in the BM aspirate. Fluorescent in situ Hybridization was negative for *BCR‐ABL1*,* PDGFRB,* and *FGFR1* rearrangements and for *CHIC2* deletion. In light with the hypothesis of PV according to the panmyelosis feature in the BM sections, 51Cr Red Cell Volume (RCV) and 125I Albumin were measured. Normal red cell and plasmatic volumes were observed. Serum erythropoietin level was in normal range, at 8.3 IU/L (normal range: 2.6‐18.5 IU/L). There was no evidence of iron deficiency. Because of a high reticulocyte count, a hemolysis screening was performed and demonstrated a decreased haptoglobin level at 0.24 g/L (normal range: 0.85‐2.30 g/L), an increased non‐conjugated bilirubin level at 58 μmol/L (normal range: <17 μmol/L) and an elevated lactate dehydrogenase level at 561 IU/L (normal range: 135‐225 IU/L), confirming a hemolytic process. To objective the hemolysis, a 51Cr red blood cells lifespan was performed, which was at the lower limit of the normal (24 days, normal range: 24‐32 days), with a moderate and compensated hyperhemolysis, without hepatosplenic fixation. The etiology of hemolysis is still unknown (absence of morphological abnormalities of the erythrocytes on the PB smear, negative direct antiglobulin test, absence of paroxysmal nocturnal hemoglobinuria clone, normal Hb electrophoresis, normal 6‐phosphoglutonate dehydrogenase and pyruvate kinase activities). Finally, taken together all these features were consistent with a diagnosis of PV masked due to a hemolytic process. Daily low‐dose acetyl salicylic acid was introduced. Neither phlebotomy nor cytoreductive therapy was necessary according to the therapeutic guidelines of PV.^7^ Eight months after the diagnosis, no thrombotic events were reported. WBC and red blood parameters remained stable and hemolysis was still observed.

**Table 1:** Mutations screened by next‐generation sequencing in the peripheral blood

Gene—exon	Result
*ASXL1* exon 12	WT
*CALR* exon 9	WT
*CBL* exon 8‐9	WT
*CBLB* exon 8‐10	WT
*CBLC* exon 8‐10	WT
*CSF3R* exon 14‐17	WT
*DNMT3A* exon 2‐223	WT
*EZH2* exon 2‐20	Mutated exon 8 p.E275Afs*12, 36%
*IDH1* exon	WT
*IDH2* exon 4	WT
*IKZF1* exon 2‐8	WT
*JAK2* exon 3‐25	Mutated exon 14 V617F, 54%
*KRAS* exon 2‐3	WT
*MPL* exon 10	WT
*NFE2* exon 3‐4	WT
*NRAS* exon 2‐5	WT
*SETBP1* exon 4	WT
*SF3B1* exon 13‐16	WT
*SH2B3* exon 2‐8	WT
*SRSF2* exon 1	WT
*U2AF1* exon 2 and 6	WT
*TET2* exon 3‐11	WT
*TP53* exon 2‐11	WT

WT, wild type.

A *JAK2* V617F mutation was detected with a high mutated allele burden at 54%. An additional *EZH2* exon 8 p.E275Afs*12 mutation was also observed.

## 3 DISCUSSION

The WHO diagnosis criteria of PV have been modified in 2016 with the decrease of Hb thresholds from 18.5 to 16.5 g/dL for male and from 18.0 to 16.0 g/dL for female and the introduction of Ht cut‐offs (49% for male and 48% for female),^3^ making the diagnosis of PV more sensitive.^8^ However, these criteria could miss a category of patients, with Hb and Ht levels below the cutoffs but meeting the other major criteria of PV. These likely correspond to the mPV category defined in view of the WHO 2008 criteria but several definitions were used for mPV and controversial results were observed between studies in terms of outcomes.^4^, ^5^, ^6^

In the WHO 2016 classification, the BM evaluation became a major criterion, highlighting its central place for the differentiation of reactive state, PV/mPV, and other MPN, especially for the discrimination between mPV and Essential Thrombocythaemia (ET).^3^, ^9^ In the case reported here, the patient presented Hb and Ht levels below the WHO 2016 cutoffs and RCV was not increased, lacking the first major criterion for the diagnosis of PV.^3^ Hb level was also below the originally proposed thresholds for the diagnosis of mPV (16.0‐18.4 g/dL for male).^4^ Nevertheless, a panmyelosis feature was observed in the BM sections and a *JAK2* V617F mutation was detected, meeting the second and the third WHO 2016 major criteria respectively.^3^ Moreover, no argument in favor of another MPN was observed: absence of fibrosis in the BM sections, absence of BCR‐ABL1 transcripts and of other mutations in the PB, except an additional *EZH2* exon 8 mutation. In the case reported here, a hemolytic process was observed rising the hypothesis of a PV masked due to hemolysis. The hemolytic process was probably responsible for normal Hb and Ht levels and normal RCV. Although masked PV due to an iron deficiency are well‐known,^10^ masked PV due to hemolysis are uncommon and this association could be undiagnosed or misdiagnosed with another MPN. Nevertheless, a differential diagnosis between early phases of PV, ET, and PMF is necessary because of their differences according to prognosis and therapeutic management.^7^ The case reported here highlights the importance of the BM biopsy to rule out the differential diagnoses and to establish an accurate diagnosis in atypical MPN blood presentations. Moreover, in the light with the high mutated allele burden of *JAK2* V617F mutation reported in this patient, we propose that a panmyelosis feature in the BM sections and/or a high allelic load of *JAK2* V617F mutation, without significant fibrosis, should conduce to perform a reticulocyte count and a hemolysis screening, despite normal Hb, Ht, or RCV levels.

Several studies reported the same probability of thrombosis between mPV and overt PV, suggesting that mPV should be diagnosed without delay and managed according to the PV guidelines to prevent thrombotic events.^4^, ^5^, ^7^ Conversely, controversial results were observed between studies in terms of risk of transformation to acute myeloid leukemia and survival.^4^, ^5^ In view of the recent revision of the WHO criteria, re‐definition and harmonization of the mPV category are mandatory to clarify its thrombosis risk and its outcome.

Though uncommon, clinicians should be aware of the association of hemolysis and PV and report of such cases could help them in clinical practice to establish an early and accurate diagnosis that could be challenging in atypical MPN presentations.

## CONFLICT OF INTEREST

None declared.

## AUTHORSHIP

All authors critically revised the manuscript and approved the final submitted version. CDE and LF: collected data and drafted the manuscript. RJ and AM: carried out the histological analysis. CDO: acquired and analyzed red blood cells data. BC: acquired and analyzed molecular data.
